# The structural basis of a high affinity ATP binding ε subunit from a bacterial ATP synthase

**DOI:** 10.1371/journal.pone.0177907

**Published:** 2017-05-18

**Authors:** Alexander Krah, Yasuyuki Kato-Yamada, Shoji Takada

**Affiliations:** 1 Department of Biophysics, Graduate School of Science, Kyoto University, Kitashirakawa-Oiwakecho, Sakyo-ku, Kyoto, Japan; 2 School of Computational Sciences, Korea Institute for Advanced Study, Dongdaemun-gu, Seoul, Republic of Korea; 3 Department of Life Science, College of Science, Rikkyo University, Nishi-Ikebukuro, Toshima-ku, Tokyo, Japan; University of Cambridge, UNITED KINGDOM

## Abstract

The ε subunit from bacterial ATP synthases functions as an ATP sensor, preventing ATPase activity when the ATP concentration in bacterial cells crosses a certain threshold. The R103A/R115A double mutant of the ε subunit from thermophilic *Bacillus* PS3 has been shown to bind ATP two orders of magnitude stronger than the wild type protein. We use molecular dynamics simulations and free energy calculations to derive the structural basis of the high affinity ATP binding to the R103A/R115A double mutant. Our results suggest that the double mutant is stabilized by an enhanced hydrogen-bond network and fewer repulsive contacts in the ligand binding site. The inferred structural basis of the high affinity mutant may help to design novel nucleotide sensors based on the ε subunit from bacterial ATP synthases.

## Introduction

ATP synthases, the universal energy conversion machinery in all living cells, synthesize ATP by a rotational motion [1], inducing the catalytic reaction of synthesizing ATP by phosphorylating ADP. This rotational motion is induced by an electrochemical proton [2] or sodium ion [3] gradient; the molecular and energetic basis for the ion selectivity have been characterized previously [4]. Vice versa, under certain cellular conditions, ATP hydrolysis can be induced to maintain the electrochemical gradient, rotating in the opposite direction [5]. To prevent the waste of ATP, ATPase activity in bacteria and mammals is supressed by common and distinct regulatory mechanisms. The common ATPase inhibition mechanism in mammals [6] and bacteria [7] is MgADP inhibition [8]. Additionally the mammalian ATP synthase is regulated by IF_1_ [9], sensing the pH in the cell. If the pH drops below 6.5 [10], the IF_1_ tetramer [11] splits into two dimers and inhibits two ATP synthases simultaneously [12]. The ATPase activity in bacterial cells is regulated by the ε subunit, sensing the ATP concentration. A large conformational change from the non-inhibitory down- to the inhibitory up-state is induced, if the ATP concentration passes a certain threshold [13–15]. This transition inhibits ATP hydrolysis activity by binding to the catalytic α_3_β_3_ subunit assembly [16–18], slowing down hydrolysis and synthesis reactions by supressing substrate binding and product release [19]. A recent review on the different regulatory mechanism of bacterial and mammalian ATP synthases can be found elsewhere [20].

For the first time it was shown in 2003 that the isolated ε subunit from thermophilic *Bacillus* PS3 binds ATP [21]. The ATP threshold of the ε subunit from bacterial ATP synthases is found from the μM (4.3 μM) in the wild type ε subunit from thermophilic *Bacillus* PS3 [22] to the mM range in the wild type ε subunit from *Bacillus subtilis* (2.1 mM) [23] or *Escherichia coli* (22 mM) [24]. The decrease in the binding affinities of the latter relative to the ε subunit from thermophilic *Bacillus* PS3 may be caused by different ATP binding residues [25] and an allosteric Mg^2+^ binding site [26] for *Escherichia coli* and *Bacillus subtilis*, respectively. Indeed, it has been shown that alanine mutations of residues located in the binding site in the ε subunit from thermophilic *Bacillus* PS3 decrease the binding affinity [22]. Surprisingly, however, the introduction of the R103A/R115A double mutation into the ε subunit from thermophilic *Bacillus* PS3 increases the binding affinity of ATP by two orders of magnitude [27]. Despite available biochemical data for some organisms, structural data clarifying the different ATP binding strengths of the ε subunit from these organisms is hardly available. The ε subunit from *Thermosynechococcus elongates* BP-1 could be resolved by NMR spectroscopy [28] and the structural basis of the ε subunit from *Escherichia coli* could be elucidated by NMR spectroscopy [29] and X-ray crystallography [25]. These structures are obtained for the isolated subunit in the down-state not being bound to ATP. In addition, crystal structures of the isolated ε subunit from thermophilic *Bacillus* PS3 [24] could give first insights into ATP binding to subunit ε. A theoretical study on the ε subunit from thermophilic *Bacillus* PS3 [30] could identify that Mg^2+^ is bound to ATP:Oα/Oβ, being confirmed recently by the structure of the ε subunit from *Caldalkalibacillus thermarum* [31]; the corresponding ATP binding site of the ε subunit from *Caldalkalibacillus thermarum* is shown in S1 Fig.

Based on the ε subunit from bacterial ATP synthases, ATP sensor proteins [32–34] have been developed recently and applied to understand biological functions for several organisms as mammals [35], plants [36], bacteria [33] and cells infected by a virus [37]. The R103A/R115A double mutant therefore has the potential to further extend the range of measuring ATP concentrations in a low concentration range, as e.g. for changes of extracellular ATP levels, important for signal transduction.

Here, we infer the structural basis of the ATP binding site of the R103A/R115A double mutant of the ε subunit from thermophilic *Bacillus* PS3, using molecular dynamics simulations and free energy calculations. In the double mutant, we observe a different binding site structure from what we have previously obtained for the wild type protein [30]. In the double mutant, an enhanced hydrogen bond network is observed and repulsive interactions between ligand coordinating residues themselves and the Mg^2+^ ion are reduced. Clarifying the structural basis of the R103A/R115A mutant, may allow to introduce additional site-directed mutations to design novel nucleotide sensors, as e.g. ADP or AMP sensors, based on the ε subunit from thermophilic *Bacillus* PS3. These sensors might be helpful to monitor the AMP/ADP concentrations to derive additional information about signalling pathways or the role of relevant proteins in diseases as e.g. the platelet ADP receptor [38] or adenosine monophosphate-activated protein kinase (AMPK) [39].

## Material and methods

### Conventional molecular dynamics simulations

**Fig 1:**
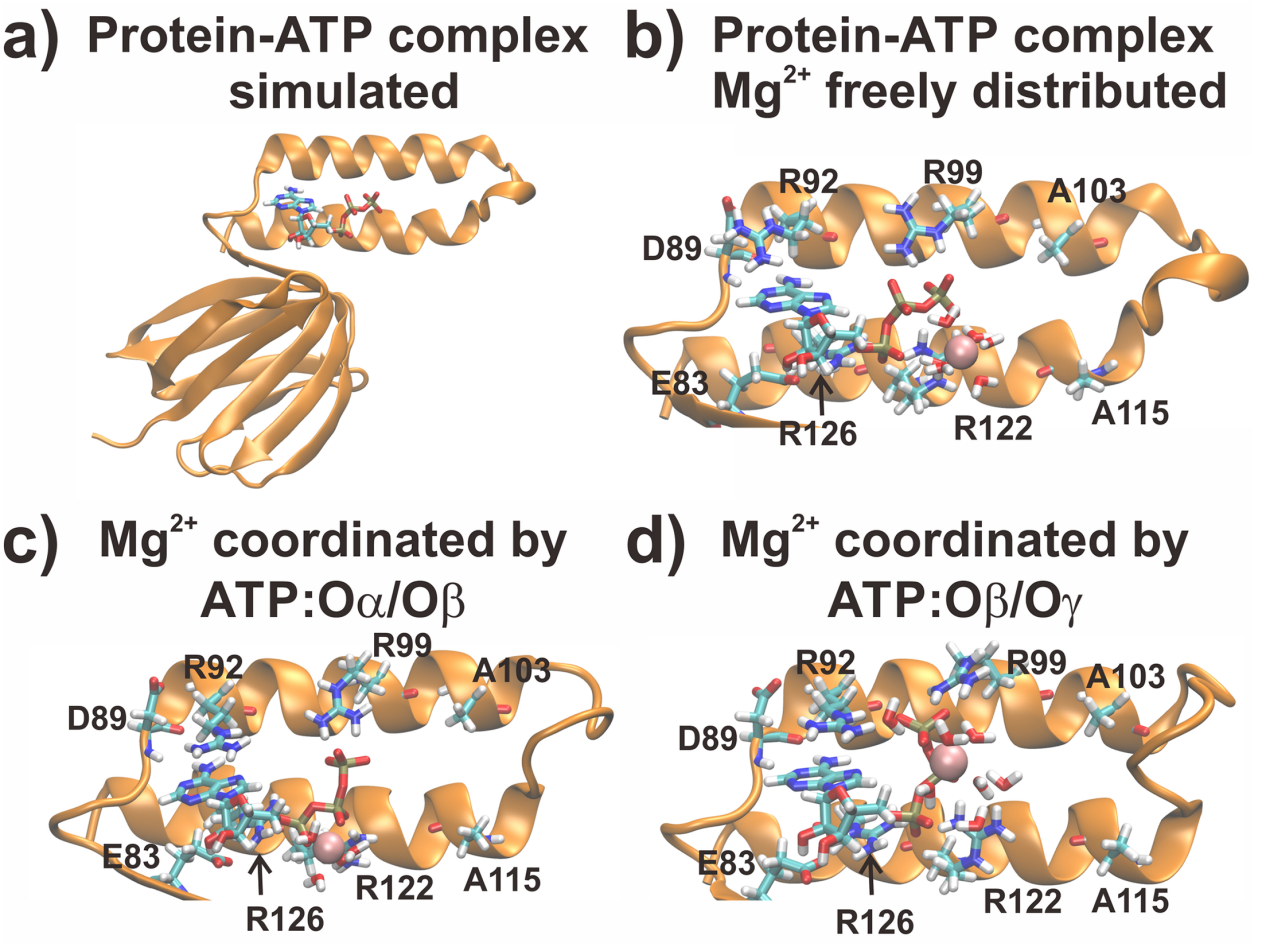
Input structures for the simulations. In a) the whole protein-ATP complex is shown. In b-d) the initial binding site structures for the freely distributed Mg^2+^ case, the MgATP:Oα/Oβ and MgATP:Oβ/Oγ coordination are shown, respectively. Water molecules coordinating the Mg^2+^ ion and residues coordinating ATP are shown in licorice, while the Mg^2+^ ion is shown in VdW spheres.

Conventional MD simulations were carried out for the R103A/R115A mutant from the ε subunit from thermophilic *Bacillus* PS3 for three different sets. Starting structures for this runs were obtained from our previous (wild type) simulation after a total simulation time without any restraints (production) of 150 ns [30], followed by introduction of the mutations (R103A and R115A), thus we already used an equilibrated structure as an input; in this initial model no Mg^2+^ ion was bound to ATP. These systems were simulated for 150 ns; four Mg^2+^ ions were freely distributed in the bulk. In the second and third set of simulations, the final structures of the initial simulation (Mg^2+^ freely distributed) were taken as the starting structure for each individual run; one Mg^2+^ ion was modelled in a first sphere coordination to ATP:Oα/Oβ or ATP:Oβ/Oγ, respectively. The whole system and all three setups, denoting a different Mg^2+^ coordination states, are shown in Fig 1, highlighting the ligands (ATP and Mg^2+^) and the binding site. The remaining three Mg^2+^ ions were freely distributed in the simulation system. These simulations were carried out for 100 ns. Three independent replicas were simulated for each system. We added counter ions to neutralize the simulation systems for all runs.

Simulations were carried out with the MD program GROMACS (version 4.6.5) [40], using the AMBER-ILDN force field [41–45], which has been implemented in GROMACS previously [46]. Mg^2+^ ion parameters were utilized as reported previously [47]. To keep pressure and temperature constant at 1 bar and 300 K, we used the Parrinello-Rahman barostat [48] and the velocity rescale thermostat [49]. Electrostatic interactions were calculated using a real space cut-off of 12 Å by the Particle Mesh Ewald method (PME) [50]. Van der Waals interactions were calculated using the same cut-off distance. An integration time step of 2 fs was used.

### Free energy calculations

To clarify the Mg^2+^ coordination sphere, we calculated the solvation free energy of an Mg^2+^ ion in a second sphere coordination (the ATP phosphate group and the Mg^2+^ ion are bound via bridging water molecules) and in two possible first sphere coordination states (Mg^2+^ is bound to either ATP:Oα/Oβ or ATP:Oβ/Oγ). The different ion coordination states by ATP are shown in Fig 1 (Fig 1b: Mg^2+^ is shown in a second sphere coordination towards ATP, c: Mg^2+^ coordinated by ATP:Oα/Oβ and d: Mg^2+^ coordinated by ATP:Oβ/Oγ, respectively). We removed all Mg^2+^ ions, not being present in the sphere of interest (not the ones bound in the second sphere or bound to ATP in a first sphere, but the remaining three ions) to exclude any attractive effects of ATP to any of these Mg^2+^ ions (other than the bound one). The thermodynamic integration (TI) method was applied. We increased the coupling factor λ incrementally, using 85 windows. Van der Waals and electrostatic interactions were removed serially without using a soft-core potential. A restraint of 1.5 kcal/(mol*Å^2^) was introduced to keep the ion in the designated position (relative position restraints of the Mg^2+^ ion to the ATP phosphate group). Forward and backward simulations were carried out for 500 ps per window, using the first 100 ps as equilibration time. Analysis of the results was carried out using the Bennet Acceptance Ratio (BAR) method [51] with the g_bar module as implemented in GROMACS. The standard deviation was derived on the basis of forward and backward direction of the TI calculations.

### MM-PBSA calculations

Molecular Mechanics–Poisson Boltzmann Surface Area (MM-PBSA) calculations [52] were carried out with the program g_mmpbsa [53]. The electrostatic potential was calculated via APBS [54]. The ε subunit (R103A/R115A double mutant) was assigned as the protein, while ATP, Mg^2+^ and the Mg^2+^-coordinating water molecules were defined as the ligand. We used the non-linear Poisson Boltzmann method (NLPB) [55]. The dielectric constant for the solute was set to ε = 2 and for the solvent ε = 80. A physiological NaCl concentration (150 mM) was added for the calculations. After an equilibration time of 10 ns, we analysed 900 snapshots of the remaining 90 ns per run and coordination state, skipping every 100 ps.

### Additional analysis

To additionally explain the experimental results, we analysed the hydrogen bond network of the protein towards ATP and the protein-protein network between the second α-helical C-terminal domain, ranging from residue 112 to residue 133, with the remaining protein, using a cut-off distance of 2.7 Å from the donor hydrogen atom to the acceptor atom and a cut-off of 30° for the angle between donor hydrogen and acceptor atoms. Estimation of the energy contribution of the hydrogen bond network, was estimated as proposed by Espinosa et al. [56]:

$$E_{HB}=-\frac{50}{2}*10^{3}*e^{\left(-3.6*d\left(H-O\right)\right)}\left[kJ/mol\right]$$

where E_HB_ denotes the hydrogen binding energy and d(H-O) represents the distance between the donor hydrogen and the acceptor atom in Å.

To allow an initial equilibration of the system, we skipped the first 10 ns in this analysis. In addition, repulsive contacts were defined as contacts of positively charged residues located in the binding site and Mg^2+^ using a maximum distance of 4.5 Å. Standard deviations were calculated using the averages of all three runs.

## Results

**Fig 2:**
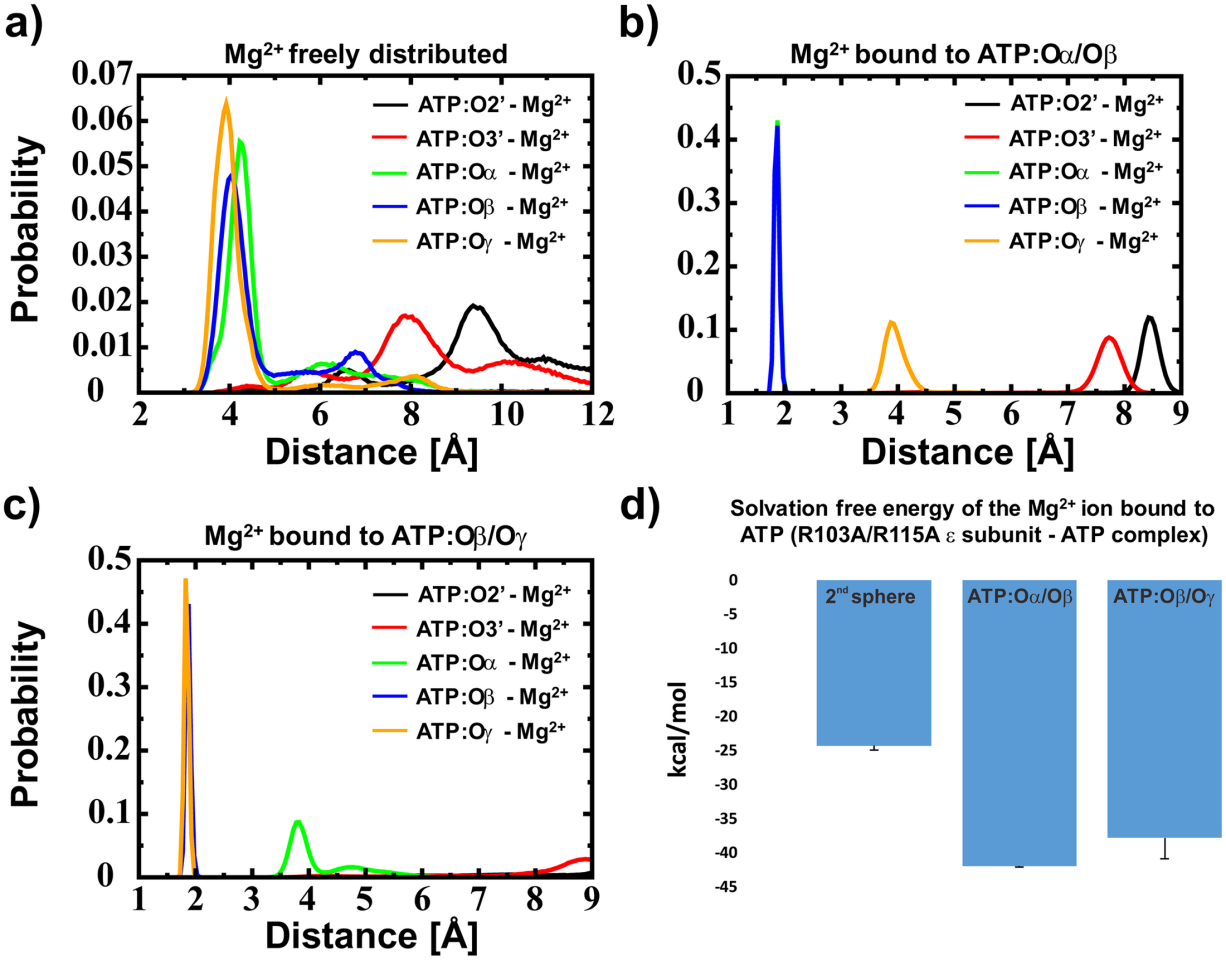
Distance distribution of the Mg^2+^ ion and free energy results. Distance distribution when the Mg^2+^ ion a) is bound in a second sphere coordination to ATP, b) is bound in a first sphere coordination to ATP:Oα/Oβ and c) is bound in a first sphere coordination to ATP:Oβ/Oγ; ATP is bound to the protein in all cases. In a)–c) the black, red, green, blue and orange lines represent the minimal distance distributions of a Mg^2+^ ion towards ATP:O2’, ATP:O3’, ATP:Oα, ATP:Oβ and ATP:Oγ, respectively. The before mentioned data were extracted from conventional MD simulations. In d) the free energy calculations (Thermodynamic Integration) for the binding of Mg^2+^ towards ATP in second sphere coordination, when bound to ATP:Oα/Oβ or ATP:Oβ/Oγ are shown, respectively. It should be noted that in Fig b) the blue and the green lines are overlapping.

Recent experiments showed an unexpected binding affinity of ATP to the ε subunit of the R103A/R115A mutant from thermophilic *Bacillus* PS3. This double mutant binds ATP two orders of magnitude stronger (K_d_ = 52 nM) [27] than the wild type (K_d_ = 4.3 μM) [22] protein. Structural reasons for these different binding strengths of the ε subunit to ATP are not obvious. To understand these experimental results, we carried out conventional MD simulations for the R103A/R115A mutant of the ε subunit from thermophilic *Bacillus* PS3. As we have previously shown for the wild type ε subunit from thermophilic *Bacillus* PS3 [30], we observe a high probability of Mg^2+^ binding in a second sphere coordination (Fig 2a).

Compared to the wild type [30], we observe higher stability in the distance distributions if Mg^2+^ is freely distributed and not bound in a first sphere to ATP (S2 Fig). However, our previously conducted simulations obtained the presence of an Mg^2+^ bound to ATP, between atoms Oα and Oβ (in the first sphere coordination) [30]. Thus, we carried out additional conventional MD simulations, showing a stable Mg^2+^ ion coordination to ATP (Fig 2b and 2c). These simulations were followed by free energy calculations with an Mg^2+^ ion bound between Oα/Oβ and between Oβ/Oγ to derive the binding site composition. The free energy calculations show that the Mg^2+^ ion is bound to ATP in a first sphere coordination complex (Fig 2d)–to test if the results are converged, we analysed two additional time windows (100 ps– 300 ps and 300 ps to 500 ps per TI window); the results are shown in S3 Fig and indicate that the results derived by the solvation free energy calculations of the Mg^2+^ ion are converged. To further derive the binding mode of the Mg^2+^ ion, we analysed the obtained data with the MM-PBSA method [52]. The results indicate that the Mg^2+^ ion is bound to the ATP:Oα/Oβ atoms. The obtained average value for MgATP binding over all three runs is -27.9 +/- 0.8 kcal/mol for the ATP:Oα/Oβ and -25.4 +/- 4.2 kcal/mol for the ATP:Oβ/Oγ Mg^2+^ bound state. It should be noted that the MM-PBSA calculations failed to derive the energy differences and even the trend in respect to the wild type protein (-91.9 kcal/mol and -88.2 kcal/mol for the Mg^2+^ ion bound to ATP:Oα/Oβ and ATP:Oβ/Oγ, respectively [30]). However, a recent study claimed that the MM-PBSA method is capable to distinguish between similar ligands bound to the same protein [57]. The hydrogen bond analysis, as shown in Table 1a), also supports that the ion is coordinated by ATP:Oα/Oβ (instead of ATP:Oβ/Oγ). Converting our simulation results into hydrogen binding energy (E_HB_), as introduced by Espinosa et al. [56], we obtained 99.8 +/- 1.8 kcal/mol and 95.1 +/- 2.6 kcal/mol for the Oα/Oβ and the Oβ/Oγ Mg^2+^ bound state, respectively, as it has been predicted previously for the wild type [30] and also has been shown by a current X-ray structure of the ε subunit from *Caldalkalibacillus thermarum* [31] (S1 Fig). The results are shown in Table 1b). These results are in agreement with the free energy calculations and the MM-PBSA calculations, thus we conclude that the Mg^2+^ ion is located between ATP:Oα/Oβ.

**Table 1:** Hydrogen-bond and energetic analysis of ATP binding to the wild type and mutant ε subunit.

a)	Wild type (kcal/mol)		R103A/R115A (kcal/mol)	
	Oα/Oβ	Oβ/Oγ	Oα/Oβ	Oβ/Oγ
h-bonds (protein-ATP)	9.38 +/- 0.48	9.45 +/- 0.09	10.4 +/- 0.60	9.74 +/- 0.47
h-bonds (2^nd^ α-helix)	5.27 +/- 0.20	4.93 +/- 0.69	4.01 +/- 0.18	4.05 +/- 0.26
repulsive contacts	1.96 +/- 0.51	1.58 +/- 0.20	1.08 +/- 0.51	1.54 +/- 0.06
b)	Wild type (kcal/mol)		R103A/R115A (kcal/mol)	
	Oα/Oβ	Oβ/Oγ	Oα/Oβ	Oβ/Oγ
E_HB_ (h-bonds (protein-ATP))	64.6 +/- 2.4	65.2 +/- 0.1	73.4 +/- 2.1	67.8 +/- 2.9
E_HB_ (h-bonds (2^nd^ α-helix))	28.6 +/- 0.7	27.0 +/- 2.3	26.4 +/- 1.5	27.4 +/- 2.3
E_HB_	93.2 +/- 0.31	92.2 +/- 0.42	99.8 +/- 1.8	95.2 +/- 2.6

a) The number of hydrogen bonds between the protein and ATP, internal protein-protein hydrogen bonds of the C-terminal second α-helix to the rest of the protein and repulsive contacts in the ε subunit from the wild type and the R103A/R115A mutant derived from MD simulations. b) Energetic analysis of the hydrogen bonding network towards ATP and the flexible α-helices. In a) and b) Oα/Oβ and Oβ/Oγ denote the ATP oxygen atoms to which the Mg^2+^ ion is coordinated during the simulation. Standard deviations were calculated using the averages of the three individual runs.

**Fig 3:**
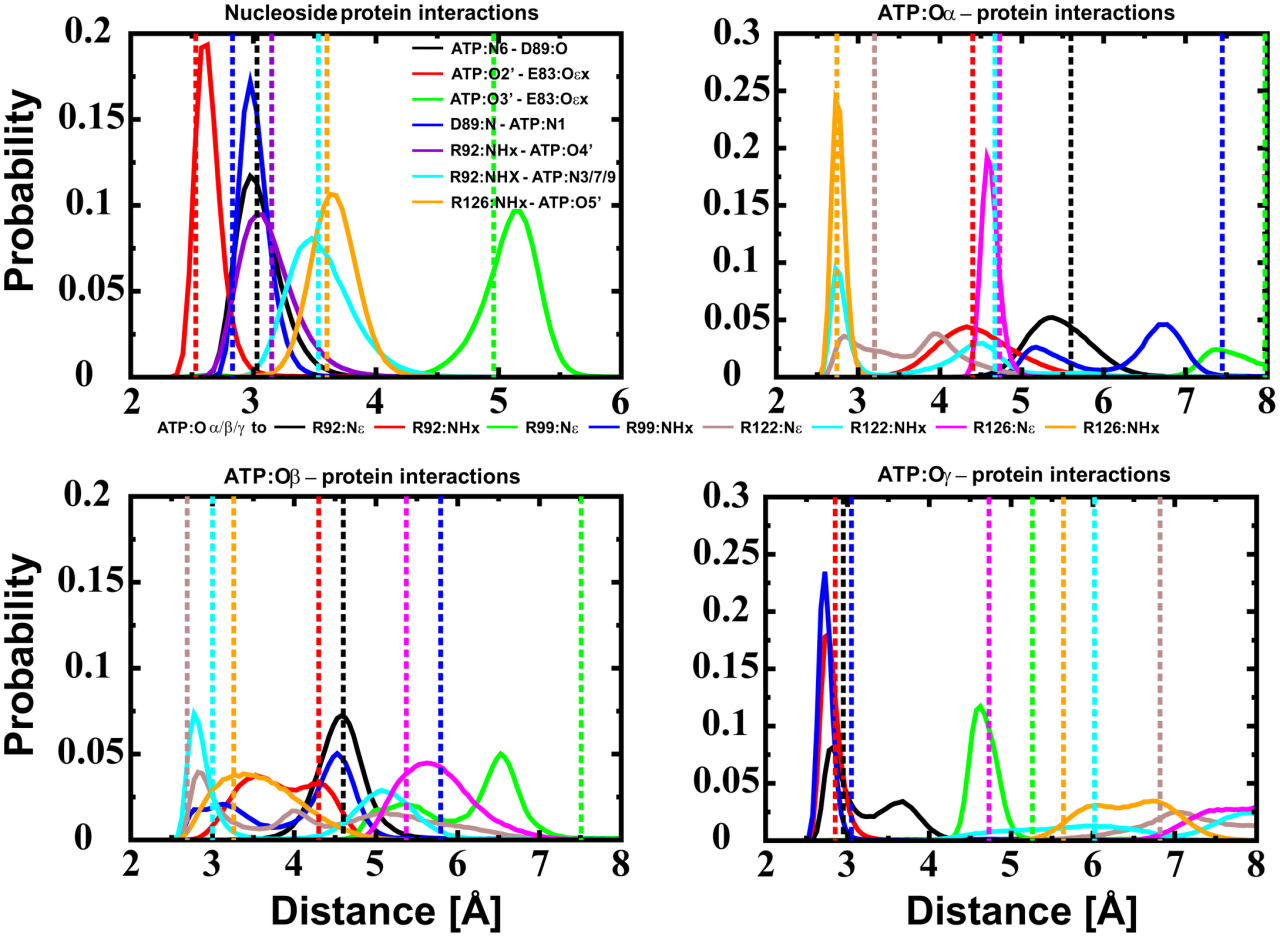
Distance distribution of protein-ATP interactions. Distance distribution of protein-ATP interactions of the Mg^2+^ bound to ATP:Oα/Oβ. Dotted lines represent distances found in the crystal structure of the wild type protein. The histogram in the top left represents nucleoside–protein interaction (black: ATP:N6 –D89:O, red: ATP:O2’–E:83:Oεx, green: ATP:O3’–E83:Oεx, blue: D89:N—ATP:N1, violet: R92:NHx—ATPO4’, cyan: R92:NHx—ATP:N3/7/9 and orange: R126:NHx—ATP:O5’). The three other diagrams represent protein—ATP:Oα/β/γ interactions (black: R92:Nε, red: R92:NHx, green: R99:Nε, blue: R99:NHx, brown: R122:Nε, cyan: R122:NHx, magenta: R126:Nε and orange: R126:NHx), respectively. The corresponding figures for the Mg^2+^ freely distributed state and Mg^2+^ coordinated to ATP:Oβ/Oγ are shown in S2 and S4 Figs in the Supporting Information, respectively. The corresponding data for the single runs is shown in S5 (Mg^2+^ not bound in first sphere), S6 (Mg^2+^ bound to ATP:Oα/Oβ) and S7 Figs (Mg^2+^ bound to ATP:Oβ/Oγ), respectively.

**Fig 4:**
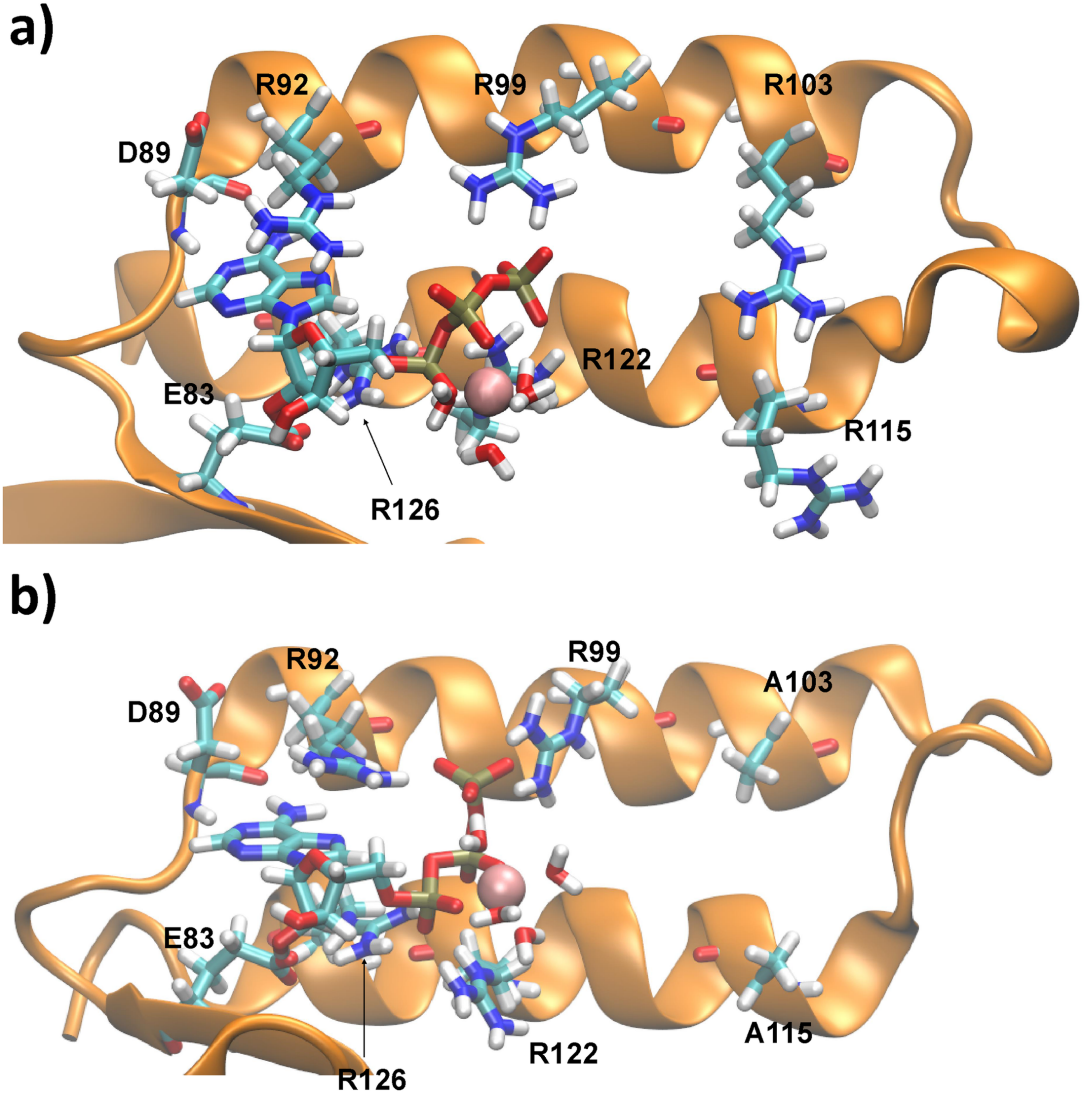
Comparison of wild type and mutant ATP binding site. ATP binding site of a representative snapshot of a) the wild type ε subunit [30] and b) the R103A/R115A double mutant derived by MD simulations. A structural comparison of the crystal structure and the R103A/R115A mutant is shown in the supporting information (S8 Fig). Figures containing molecular information have been produced using VMD [58].

The nucleoside interactions are stable in our simulations as observed in the crystal structure. Additionally, ATP:Oα is coordinated by R126:NHx and R122:NHx, ATP:Oβ by flexible interactions of R122:NHx and R126:NHx and ATP:Oγ by R92:NHx, R92:Nε and R99:NHx (Fig 3). An Mg^2+^ ion is found to be coordinated by ATP:Oα/Oβ (Fig 2d). It should be noted that R99 and R122 are slightly displaced compared to the crystal structure, representing a more favourable hydrogen bond network. The binding sites of the wild type and the mutant ε subunit derived by molecular simulations are shown in Fig 4.

To understand the different binding strength of ATP binding towards the ε subunit of the wild type [22] and the R103A/R115A mutant [27] from thermophilic *Bacillus* PS3, we analysed the hydrogen bond network of the protein towards ATP, the internal protein-protein hydrogen bond network and the repulsive contacts in the wild type and the R103A/R115A double mutant, respectively. The results are shown in Table 1. We observe an extended hydrogen binding network towards ATP in the mutant, while the hydrogen bonds between the flexible α-helix and the remaining residues are slightly reduced. To estimate the energy of hydrogen binding of MgATP to the ε subunit of thermophilic *Bacillus* PS3 (wild type and R103A/R115A mutant), we used an energy estimation based on the hydrogen binding network between the protein and the ligand [56]. Additionally, we estimated the hydrogen binding energy of the flexible α-helical domain, which undergoes the large conformational change from the down- to the up-state after ATP release. In total we calculate an energy penalty of 6.6 kcal/mol comparing the differences for the calculated hydrogen binding energy (Table 1b; ΔE_HB_ = E_HB_(mutant)–E_HB_(wild type)) of the wild type in respect to the R103A/R115A double mutant.

## Discussion

In order to explain the increased ATP binding ability of the R103A/R115A mutant [27] in respect to the wild type ε subunit from thermophilic *Bacillus* PS3 [22], we carried out MD simulations of the R103A/R115A double mutant and compared these results with previously discussed ones of the wild type protein [30]. The ATP molecule, bound to the double mutant, binds a Mg^2+^ ion in the ATP:Oα/Oβ position, as we predicted for the wild-type; this Mg^2+^ position also could be shown by a current crystal structure of the F_1_-complex from *Caldalkalibacillus thermarum* (PDB-ID: 5HKK), harbouring the ε subunit in the ATP bound down-state [31]; ATP is coordinated by homologous residues of subunit ε in this crystal structure [31] (S1 Fig). Our data show that the R103A/R115A mutant stabilizes ATP binding by an extended hydrogen-bond network between the protein and the ATP molecule and the flexible α-helical domain (Table 1) leading to an energy penalty of 6.6 kcal/mol of ATP binding in the wild type compared to the double mutant (Table 1). It should be noted that the E83:Oεx—ATP:O2’ and protein-ATP:Oγ interactions are remarkably stabilized (Fig 3 top left—red line, bottom right and previous results [30]); the enhanced instability of this interaction in the wild type protein might be induced by attractive interactions of R103 and/or R115 with ATP:Oγ, causing a break of the ideal hydrogen-bond network—however additional biochemical/biophysical experiments and molecular dynamics simulations are necessary to understand the role of these residues in the ATP release mechanism. Assuming that the contribution of hydrophobic interactions and entropic factors are similar in the wild type and the mutant, we propose that fewer repulsive contacts of the double mutant induce a more stable binding site structure and enhance the ATP binding strength (Table 1). Taken together, all these data explain the remarkably increased (two orders of magnitudes) ATP binding affinity to the R103A/R115A double mutant [27] of the ε subunit from thermophilic *Bacillus* PS3 in respect to the wild type protein [22]. It should be noted that the model derived by X-ray crystallographic [24] for the wild type ATP binding site of the ε subunit from thermophilic *Bacillus* PS3 differs in its biological relevant monomeric state, likely caused by protein interactions (K114 and R115) of a second monomer binding to ATP of the first monomer [30]. However, the R103A/R115A mutant shows a similar binding network towards the nucleoside and phosphate group, as observed in the experimentally derived dimeric crystal structure [24], and thus represents the binding site as found in the experimentally derived structure. However, changes in the conformations of R99 and R122 can be observed. These differences are most likely caused by the presence of unfavourable protein—protein interactions in the homodimer between positively charged residues in chain A and B; newly defined interactions between R99 and R122 towards the phosphate group, repulsive interactions between K114 and/or R115 or crystal packing effects might change the conformations of R92 and R99 in the crystal structure. A structural comparison of the R103A/R115A double mutant and the wild type crystal structure of the ε subunit from thermophilic *Bacillus* PS3 is shown in S8 Fig. The charge balance is slightly changed in the R103A/R115A mutant by an Mg^2+^ ion (interacting with two phosphate oxygen atoms) bound between ATP:Oα/Oβ and the reorientation of R99 and R122, substituting the interactions with K114 and R115 as observed in the wild type homodimer.

It has been shown previously that the ε subunit from bacterial ATP synthases can be used to sense physiological ATP concentrations in real time [32–34]. ATP binds with a two orders of magnitude decreased affinity to the wild type ε subunit from thermophilic *Bacillus* PS3 (4.3 μM) [22] in respect to the R103A/R115A double mutant (52 nM) [27]. The structural basis of high affinity ATP binding to the R103A/R115A double mutant, induced by an enhanced hydrogen bond network and reduced repulsive contacts, as discussed in this study, may allow to design novel nucleotide sensors, as e.g. AMP or ADP sensors, based on the ε subunit from bacterial ATP synthases. Introducing site directed mutations to the ATP binding site and its vicinity, taking into account the protein-ligand coordination, protein-protein interactions and repulsive contacts may help to predict/design these sensors.

## Supporting information

S1 Fig In a) the structure of the whole ε subunit from *Caldalkalibacillus thermarum* is shown. In b) the ATP binding site of the same ε subunit is highlighted. ATP is coordinated by E83, D89, R92, R99, R123 and R127. Interactions with I103 and R116 cannot be observed in the crystal structure (PDB-ID: 5HKK).
(TIF)

S2 Fig Distance distribution of protein-ATP interactions of the ε subunit of the R103A/R115A double mutant from thermophilic *Bacillus* PS3 when the Mg^2+^ ion is freely distributed, not being bound to ATP in a first sphere coordination. Dotted lines represent distances found in the crystal structure of the wild type protein. The histogram in the top left represents nucleoside–protein interaction (black: ATP:N6 –D89:O, red: ATP:O2’–E:83:Oεx, green: ATP:O3’–E83:Oεx, blue: D89:N—ATP:N1, violet: R92:NHx—ATPO4’, cyan: R92:NHx—ATP:N3/7/9 and orange: R126:NHx—ATP:O5’). The three other histograms represent protein—ATP:Oα/β/γ interactions (black: R92:Nε, red: R92:NHx, green: R99:Nε, blue: R99:NHx, brown: R122:Nε, cyan: R122:NHx, magenta: R126:Nε and orange: R126:NHx), respectively.
(TIF)

S3 Fig Convergence of the free energy results. The solvation free energies of the Mg^2+^ ion bound in a second sphere coordination to ATP(blue), bound to ATP:Oα/Oβ (orange) and ATP:Oβ/Oγ (grey) for different time ensembles is shown. The calculated free energy differences and the standard deviation is similar in all three time ensembles. All calculations were carried out for the protein-ATP complex.
(TIF)

S4 Fig Distance distribution of protein-ATP interactions for the R103A/R115A mutant of the ε subunit from thermophilic *Bacillus* PS3, when the Mg^2+^ ion is bound to ATP:Oβ/Oγ. Dotted lines represent distances found in the crystal structure of the wild type protein. The histogram in the top left represents nucleoside–protein interaction (black: ATP:N6 –D89:O, red: ATP:O2’–E:83:Oεx, green: ATP:O3’–E83:Oεx, blue: D89:N—ATP:N1, violet: R92:NHx—ATPO4’, cyan: R92:NHx—ATP:N3/7/9 and orange: R126:NHx—ATP:O5’). The three other histograms represent protein—ATP:Oα/β/γ interactions (black: R92:Nε, red: R92:NHx, green: R99:Nε, blue: R99:NHx, brown: R122:Nε, cyan: R122:NHx, magenta: R126:Nε and orange: R126:NHx), respectively.
(TIF)

S5 Fig Distance distribution of protein-ATP interactions of the ε subunit of the R103A/R115A double mutant from thermophilic *Bacillus* PS3 when the Mg^2+^ ion is freely distributed, not being bound to ATP in a first sphere coordination for all three individual runs. Dotted lines represent distances found in the crystal structure of the wild type protein. The histogram in the top left represents nucleoside–protein interaction (black: ATP:N6 –D89:O, red: ATP:O2’–E:83:Oεx, green: ATP:O3’–E83:Oεx, blue: D89:N—ATP:N1, violet: R92:NHx—ATPO4’, cyan: R92:NHx—ATP:N3/7/9 and orange: R126:NHx—ATP:O5’). The three other histograms represent protein—ATP:Oα/β/γ interactions (black: R92:Nε, red: R92:NHx, green: R99:Nε, blue: R99:NHx, brown: R122:Nε, cyan: R122:NHx, magenta: R126:Nε and orange: R126:NHx), respectively.
(TIF)

S6 Fig Distance distribution of protein-ATP interactions of the ε subunit of the R103A/R115A double mutant from thermophilic *Bacillus* PS3 when the Mg^2+^ ion coordinated by ATP:Oα/Oβ in a first sphere for all three individual runs. Dotted lines represent distances found in the crystal structure of the wild type protein. The histogram in the top left represents nucleoside–protein interaction (black: ATP:N6 –D89:O, red: ATP:O2’–E:83:Oεx, green: ATP:O3’–E83:Oεx, blue: D89:N—ATP:N1, violet: R92:NHx—ATPO4’, cyan: R92:NHx—ATP:N3/7/9 and orange: R126:NHx—ATP:O5’). The three other histograms represent protein—ATP:Oα/β/γ interactions (black: R92:Nε, red: R92:NHx, green: R99:Nε, blue: R99:NHx, brown: R122:Nε, cyan: R122:NHx, magenta: R126:Nε and orange: R126:NHx), respectively.
(TIF)

S7 Fig Distance distribution of protein-ATP interactions of the ε subunit of the R103A/R115A double mutant from thermophilic *Bacillus* PS3 when the Mg^2+^ ion is coordinated by ATP:Oβ/Oγ in a first sphere for all three individual runs. Dotted lines represent distances found in the crystal structure of the wild type protein. The histogram in the top left represents nucleoside–protein interaction (black: ATP:N6 –D89:O, red: ATP:O2’–E:83:Oεx, green: ATP:O3’–E83:Oεx, blue: D89:N—ATP:N1, violet: R92:NHx—ATPO4’, cyan: R92:NHx—ATP:N3/7/9 and orange: R126:NHx—ATP:O5’). The three other histograms represent protein—ATP:Oα/β/γ interactions (black: R92:Nε, red: R92:NHx, green: R99:Nε, blue: R99:NHx, brown: R122:Nε, cyan: R122:NHx, magenta: R126:N≈ and orange: R126:NHx), respectively.
(TIF)

S8 Fig a) ATP binding site of the dimeric wild type ε subunit derived from the crystal structure (PDB-ID: 2E5Y), where ATP (chain A) is coordinated by K114 and R115 from chain B. b) ATP binding site of the R103A/R115A mutant derived from simulations. c) Aligned structure of the ATP binding site of the ε subunit from thermophilic *Bacillus* PS3 wild type (monomer A and B are shown in blue and red, respectively), as resolved in the crystal structure, and the R103A/R115A mutant (orange). The corresponding ATP molecules are coloured green (wild type) and violet (R103A/R115A mutant). The Mg^2+^ ion (R103A/R115A mutant) is shown in van der Waals spheres. Water molecules are omitted for clarity.
(TIF)
