# Supplementary figures and images for: The structural basis of a high affinity ATP binding ε subunit from a bacterial ATP synthase
Source: PLoS One. 2017 May 18;12(5):e0177907. doi: 10.1371/journal.pone.0177907 (PMC5436830; doi:10.1371/journal.pone.0177907)

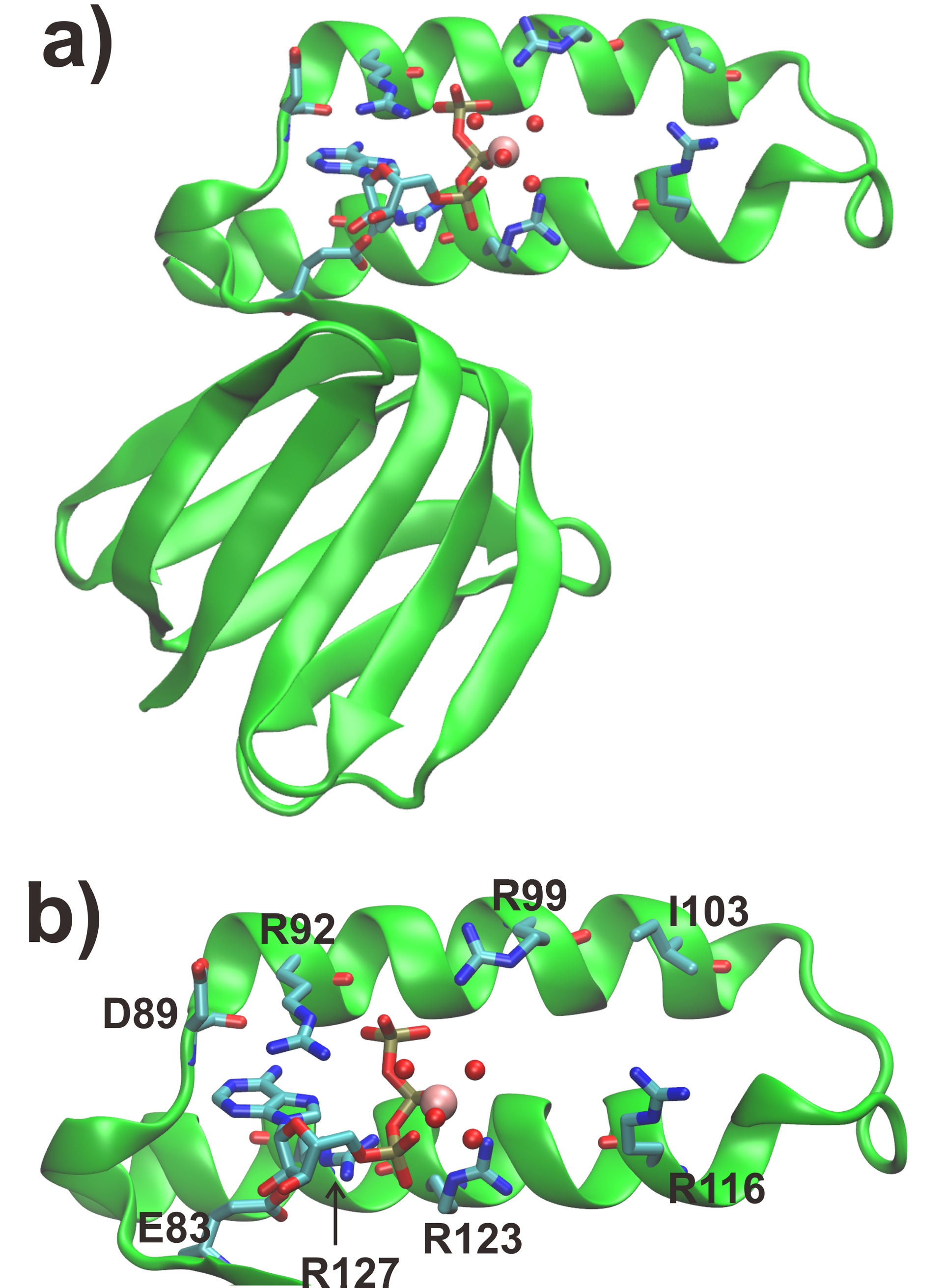

Supplement: S1 Fig — In a) the structure of the whole ε subunit from Caldalkalibacillus thermarum is shown. In b) the ATP binding site of the same ε subunit is highlighted. ATP is coordinated by E83, D89, R92, R99, R123 and R127. Interactions with I103 and R116 cannot be observed in the crystal structure (PDB-ID: 5HKK). (TIF) [file pone.0177907.s001.tif]

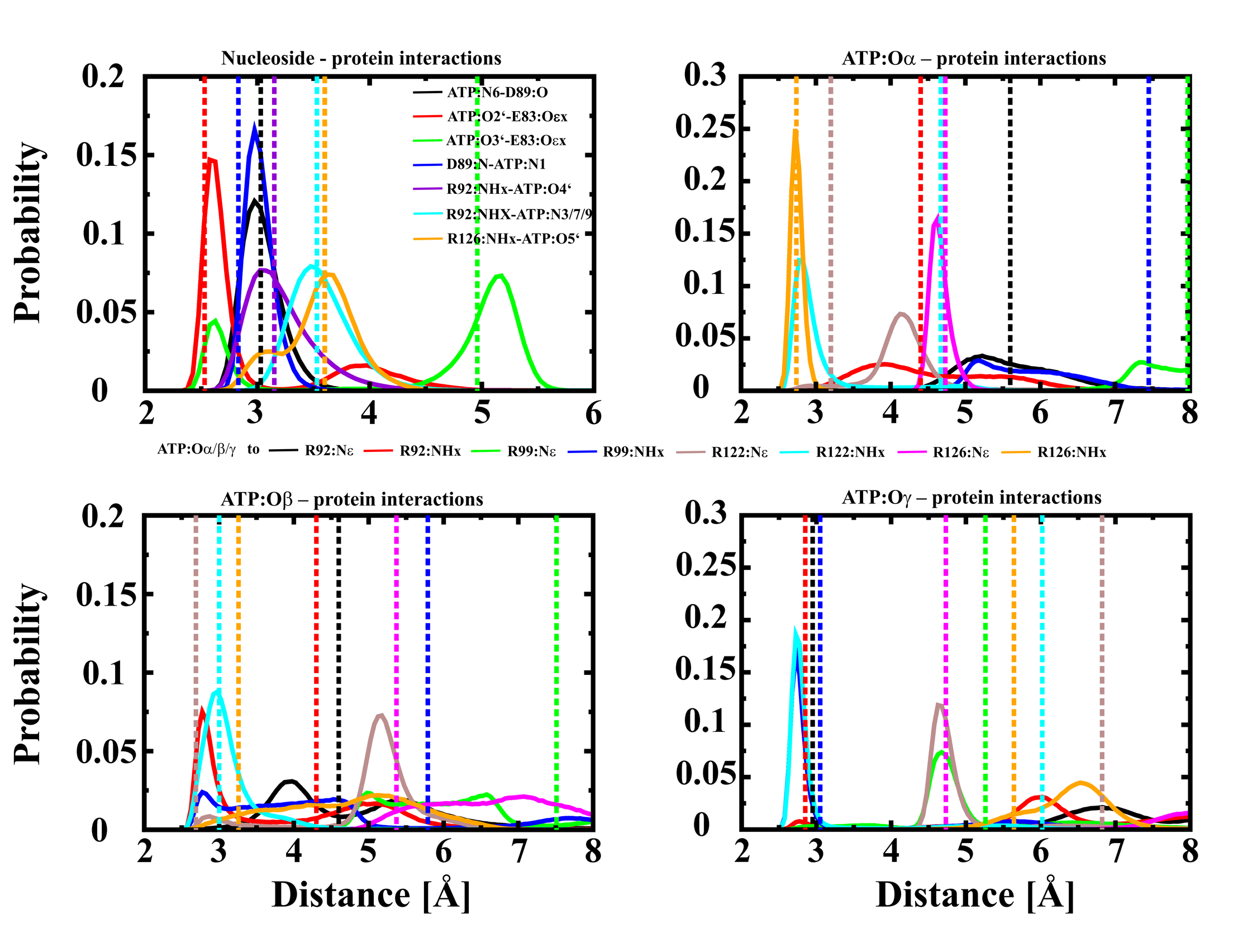

Supplement: S2 Fig — Dotted lines represent distances found in the crystal structure of the wild type protein. The histogram in the top left represents nucleoside–protein interaction (black: ATP:N6 –D89:O, red: ATP:O2’–E:83:Oεx, green: ATP:O3’–E83:Oεx, blue: D89:N—ATP:N1, violet: R92:NHx—ATPO4’, cyan: R92:NHx—ATP:N3/7/9 and orange: R126:NHx—ATP:O5’). The three other histograms represent protein—ATP:Oα/β/γ interactions (black: R92:Nε, red: R92:NHx, green: R99:Nε, blue: R99:NHx, brown: R122:Nε, cyan: R122:NHx, magenta: R126:Nε and orange: R126:NHx), respectively. (TIF) [file pone.0177907.s002.tif]

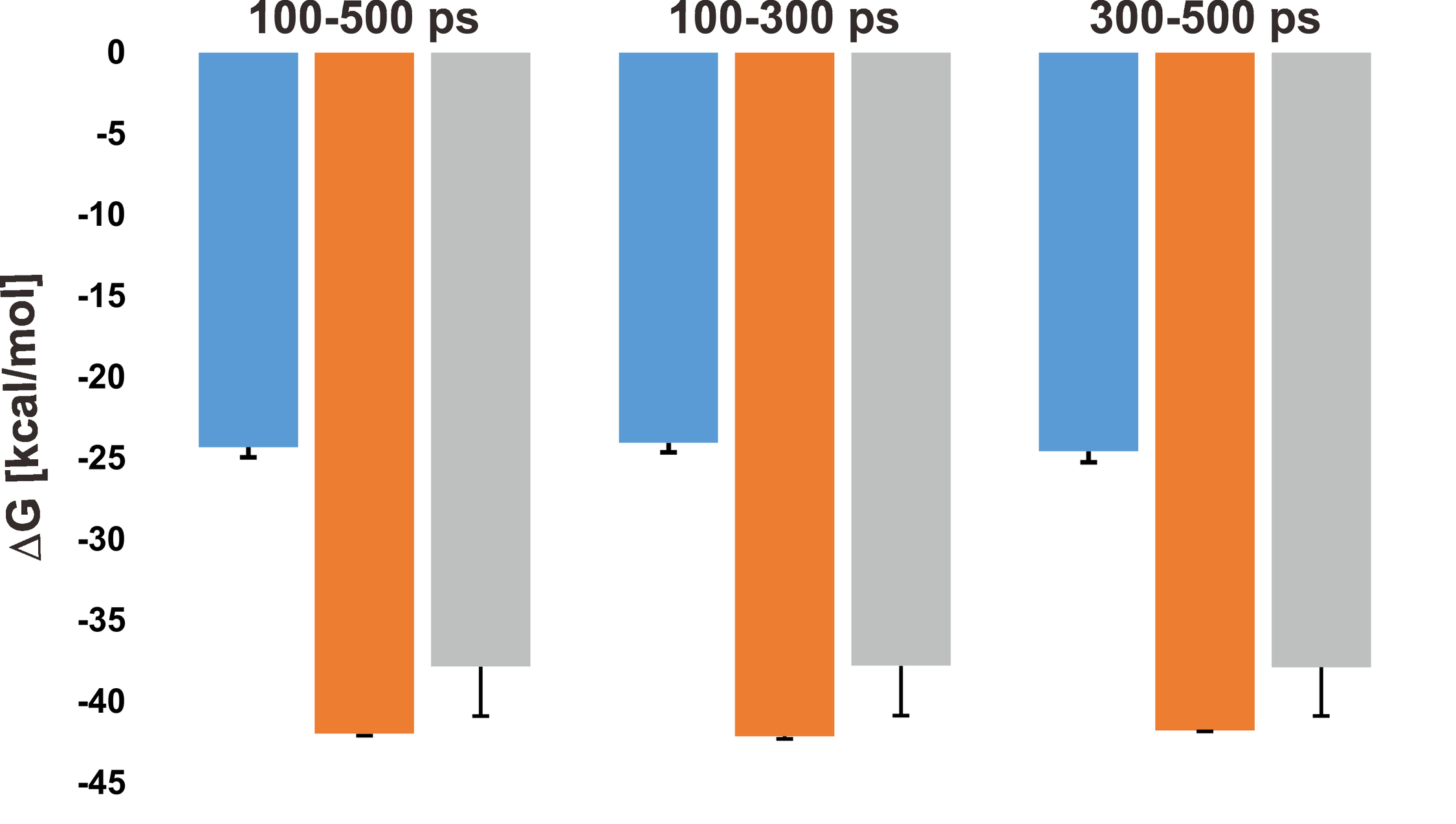

Supplement: S3 Fig — The solvation free energies of the Mg2+ ion bound in a second sphere coordination to ATP(blue), bound to ATP:Oα/Oβ (orange) and ATP:Oβ/Oγ (grey) for different time ensembles is shown. The calculated free energy differences and the standard deviation is similar in all three time ensembles. All calculations were carried out for the protein-ATP complex. (TIF) [file pone.0177907.s003.tif]

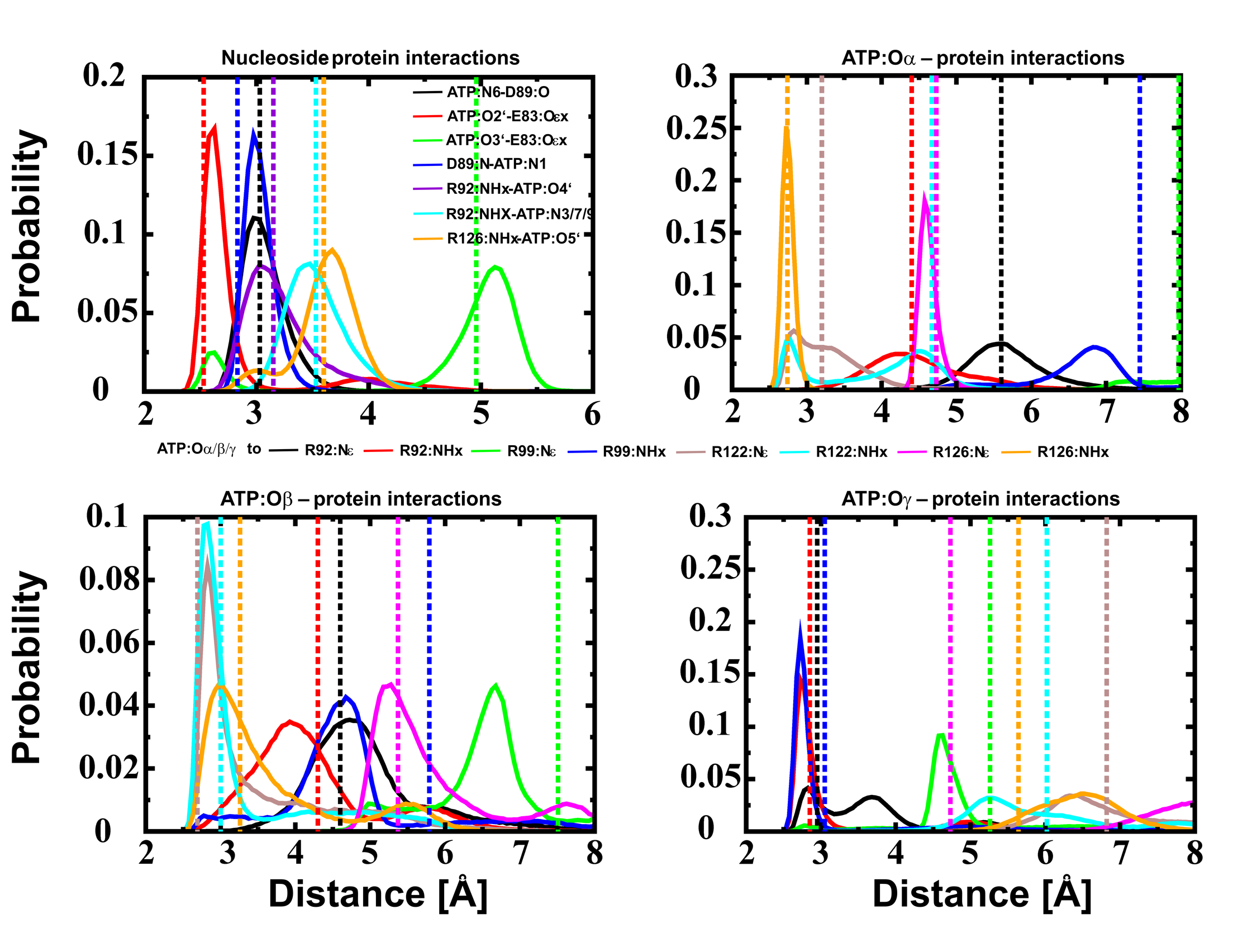

Supplement: S4 Fig — Dotted lines represent distances found in the crystal structure of the wild type protein. The histogram in the top left represents nucleoside–protein interaction (black: ATP:N6 –D89:O, red: ATP:O2’–E:83:Oεx, green: ATP:O3’–E83:Oεx, blue: D89:N—ATP:N1, violet: R92:NHx—ATPO4’, cyan: R92:NHx—ATP:N3/7/9 and orange: R126:NHx—ATP:O5’). The three other histograms represent protein—ATP:Oα/β/γ interactions (black: R92:Nε, red: R92:NHx, green: R99:Nε, blue: R99:NHx, brown: R122:Nε, cyan: R122:NHx, magenta: R126:Nε and orange: R126:NHx), respectively. (TIF) [file pone.0177907.s004.tif]

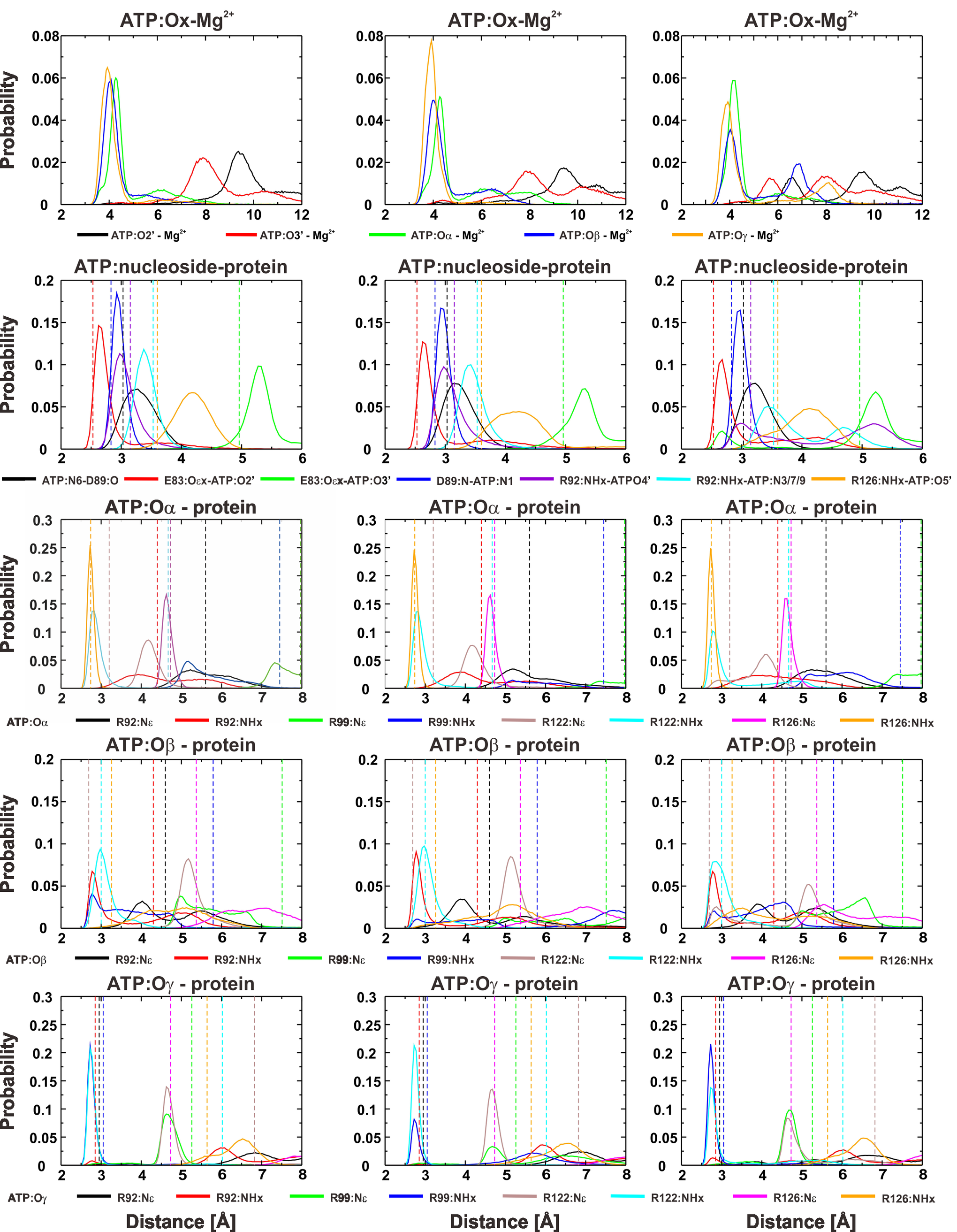

Supplement: S5 Fig — Dotted lines represent distances found in the crystal structure of the wild type protein. The histogram in the top left represents nucleoside–protein interaction (black: ATP:N6 –D89:O, red: ATP:O2’–E:83:Oεx, green: ATP:O3’–E83:Oεx, blue: D89:N—ATP:N1, violet: R92:NHx—ATPO4’, cyan: R92:NHx—ATP:N3/7/9 and orange: R126:NHx—ATP:O5’). The three other histograms represent protein—ATP:Oα/β/γ interactions (black: R92:Nε, red: R92:NHx, green: R99:Nε, blue: R99:NHx, brown: R122:Nε, cyan: R122:NHx, magenta: R126:Nε and orange: R126:NHx), respectively. (TIF) [file pone.0177907.s005.tif]

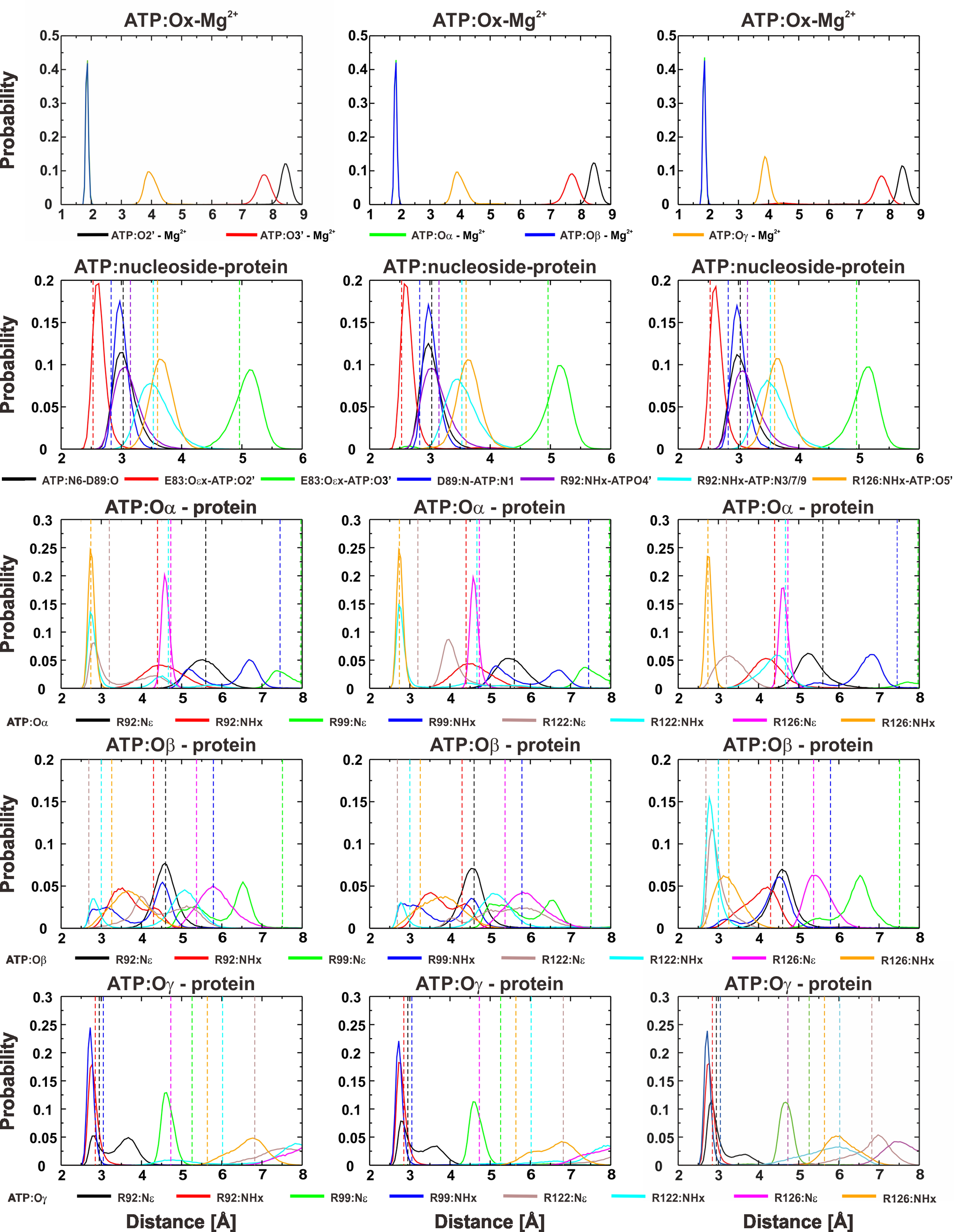

Supplement: S6 Fig — Dotted lines represent distances found in the crystal structure of the wild type protein. The histogram in the top left represents nucleoside–protein interaction (black: ATP:N6 –D89:O, red: ATP:O2’–E:83:Oεx, green: ATP:O3’–E83:Oεx, blue: D89:N—ATP:N1, violet: R92:NHx—ATPO4’, cyan: R92:NHx—ATP:N3/7/9 and orange: R126:NHx—ATP:O5’). The three other histograms represent protein—ATP:Oα/β/γ interactions (black: R92:Nε, red: R92:NHx, green: R99:Nε, blue: R99:NHx, brown: R122:Nε, cyan: R122:NHx, magenta: R126:Nε and orange: R126:NHx), respectively. (TIF) [file pone.0177907.s006.tif]

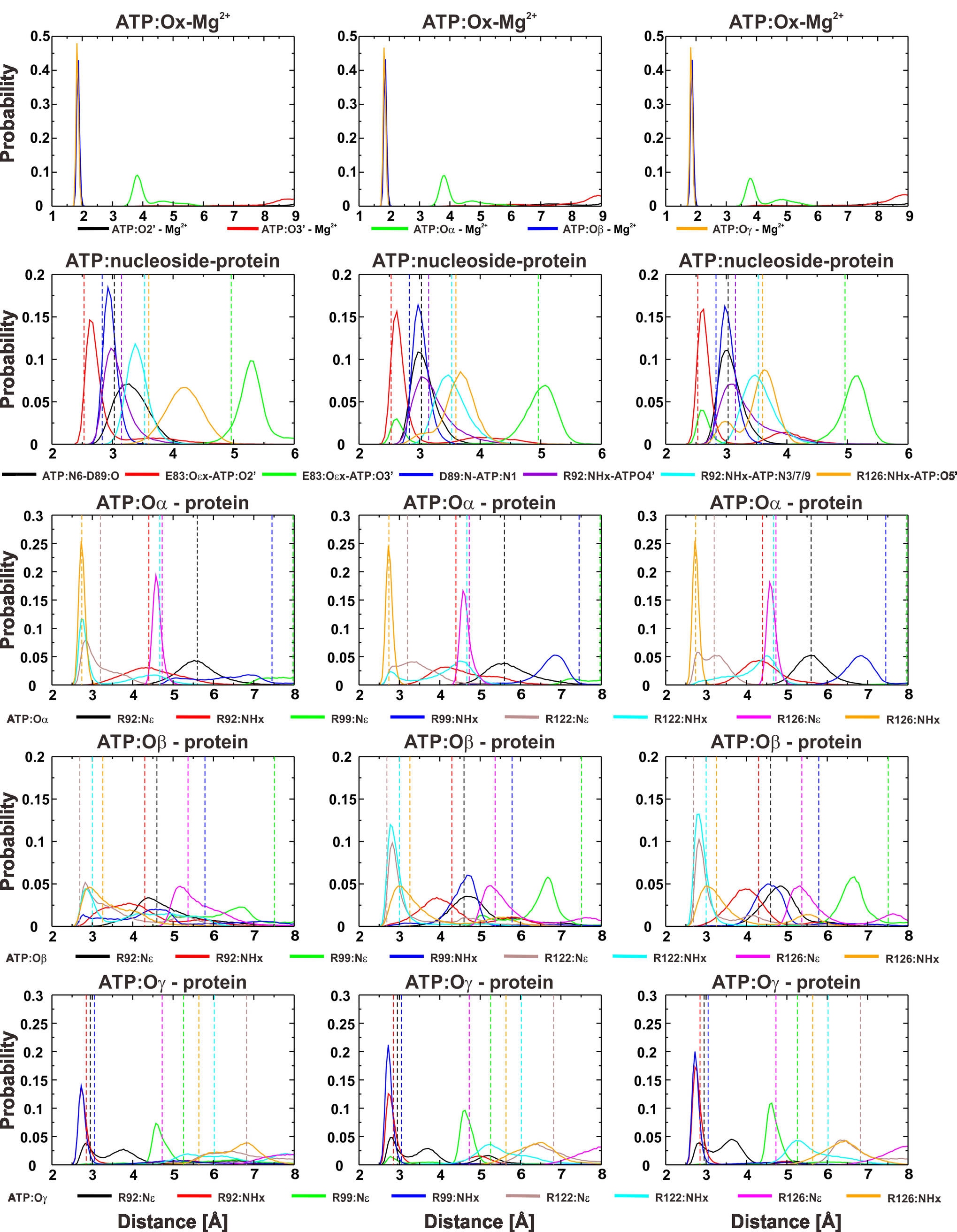

Supplement: S7 Fig — Dotted lines represent distances found in the crystal structure of the wild type protein. The histogram in the top left represents nucleoside–protein interaction (black: ATP:N6 –D89:O, red: ATP:O2’–E:83:Oεx, green: ATP:O3’–E83:Oεx, blue: D89:N—ATP:N1, violet: R92:NHx—ATPO4’, cyan: R92:NHx—ATP:N3/7/9 and orange: R126:NHx—ATP:O5’). The three other histograms represent protein—ATP:Oα/β/γ interactions (black: R92:Nε, red: R92:NHx, green: R99:Nε, blue: R99:NHx, brown: R122:Nε, cyan: R122:NHx, magenta: R126:N≈ and orange: R126:NHx), respectively. (TIF) [file pone.0177907.s007.tif]

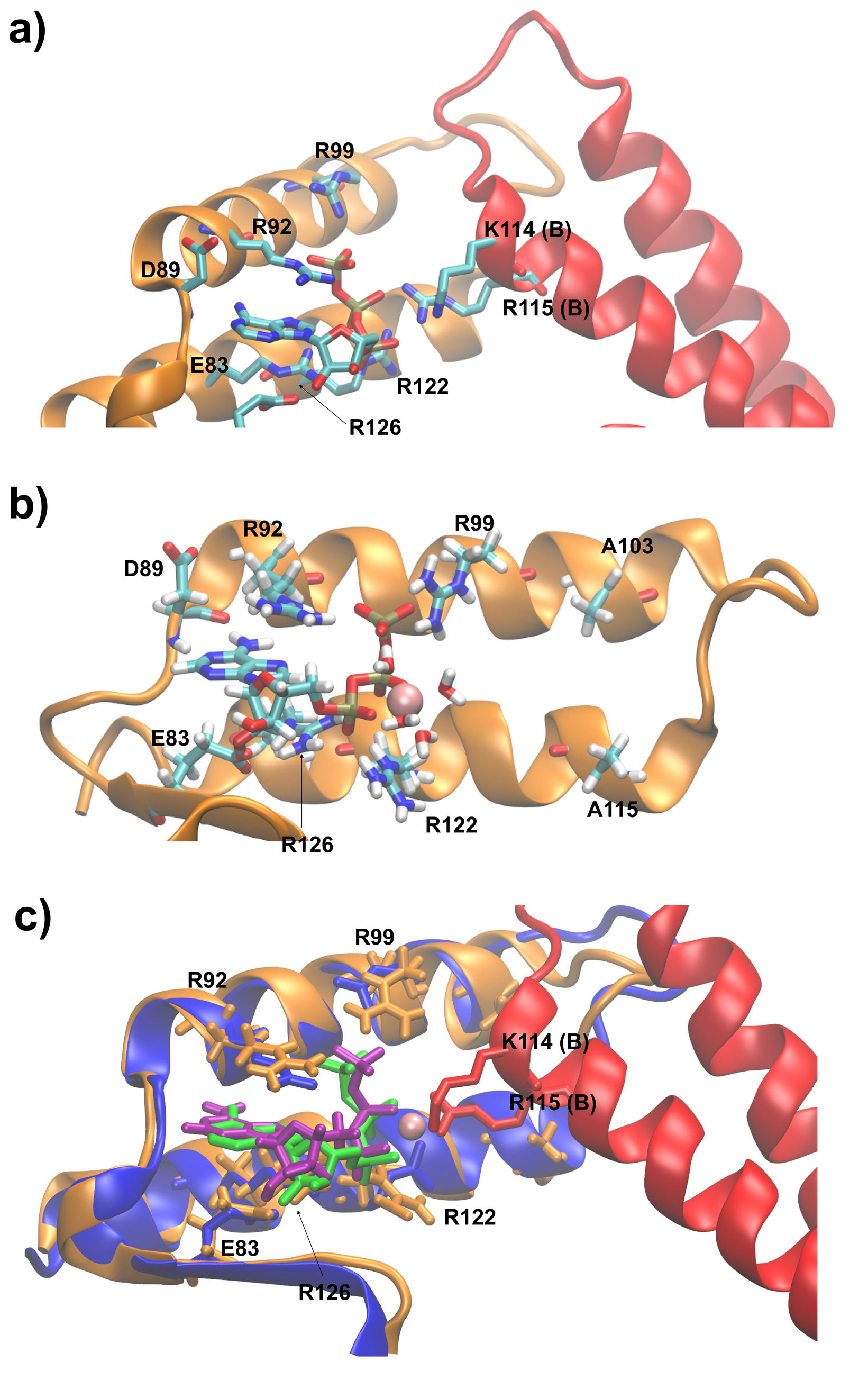

Supplement: S8 Fig — a) ATP binding site of the dimeric wild type ε subunit derived from the crystal structure (PDB-ID: 2E5Y), where ATP (chain A) is coordinated by K114 and R115 from chain B. b) ATP binding site of the R103A/R115A mutant derived from simulations. c) Aligned structure of the ATP binding site of the ε subunit from thermophilic Bacillus PS3 wild type (monomer A and B are shown in blue and red, respectively), as resolved in the crystal structure, and the R103A/R115A mutant (orange). The corresponding ATP molecules are coloured green (wild type) and violet (R103A/R115A mutant). The Mg2+ ion (R103A/R115A mutant) is shown in van der Waals spheres. Water molecules are omitted for clarity. (TIF) [file pone.0177907.s008.tif]
